# Mobility test to assess functional vision in dark-adapted patients with Leber congenital amaurosis

**DOI:** 10.1186/s12886-022-02475-y

**Published:** 2022-06-14

**Authors:** Alejandro J. Roman, Artur V. Cideciyan, Vivian Wu, Abraham A. Mascio, Arun K. Krishnan, Alexandra V. Garafalo, Samuel G. Jacobson

**Affiliations:** Scheie Eye Institute, Perelman School of Medicine, University of Pennsylvania, 51 North 39th St, PA 19104 Philadelphia, USA

**Keywords:** Childhood blindness, Cone vision, Inherited retinal degenerations, Low vision, Outcome measures, Rod vision

## Abstract

**Background:**

Inherited retinal degenerations (IRDs) affect daylight and night vision to different degrees. In the current work, we devise a method to quantify mobility under dark-adapted conditions in patients with severe childhood blindness due to Leber congenital amaurosis (LCA). Mobility thresholds from two different LCA genotypes are compared to dark-adapted vision measurements using the full-field stimulus test (FST), a conventional desktop outcome measure of rod vision.

**Methods:**

A device consisting of vertical LED strips on a plane resembling a beaded curtain was programmed to produce a rectangular pattern target defining a ‘door’ of varying luminance that could appear at one of three positions. Mobility performance was evaluated by letting the subject walk from a fixed starting position ~ 4 m away from the device with instructions to touch the door. Success was defined as the subject touching within the ‘door’ area. Ten runs were performed and the process was repeated for different levels of luminance. Tests were performed monocularly in dark-adapted and dilated eyes. Results from LCA patients with the *GUCY2D* and *CEP290* genotypes and normal subjects were analyzed using logistic regression to estimate the mobility threshold for successful navigation. The relation of thresholds for mobility, FST and visual acuity were quantified using linear regression.

**Results:**

Normal subjects had mobility thresholds near limits of dark-adapted rod vision. *GUCY2D*-LCA patients had a wide range of mobility thresholds from within 1 log of normal to greater than 8 log abnormal. *CEP290*-LCA patients had abnormal mobility thresholds that were between 5 and 6 log from normal. Sensitivity loss estimates using FST related linearly to the mobility thresholds which were not correlated with visual acuity.

**Conclusions:**

The mobility task we developed can quantify functional vision in severely disabled patients with LCA. Taken together with other outcome measures of rod and cone photoreceptor-mediated vision, dark-adapted functional vision should provide a more complete understanding of the natural history and effects of treatment in patients with LCA.

**Supplementary Information:**

The online version contains supplementary material available at 10.1186/s12886-022-02475-y.

## Background

Clinical methods to measure light-adapted cone-based vision are common and traditional [1]; less so are assays for rod-based vision. Among the methods for measuring rod function are the scotopic electroretinogram, and dark-adapted psychophysical thresholds with perimetry or a full-field stimulus test [2–6]. Techniques have also been developed to assay functional vision with mobility performed under dim light conditions where both rods and cones can be active (for example, [7–13]).

We previously developed an LED-based orientation task for measuring cone-based functional vision [14]. In the present study, the technique was modified and simplified from an orientation-only task to a mobility assay to enable quantitation of dark-adapted functional vision. Results in a cohort of patients with the childhood blindness, Leber congenital amaurosis (LCA),were compared with those in the same patients using the full-field stimulus test (FST), a psychophysical measure of visual thresholds performed with the dark-adapted subject seated with their head positioned in a ganzfeld stimulator [6, 15, 16].

## Methods

### Subjects

Subjects with healthy vision (*n* = 4; ages 22–37) and patients with *GUCY2D*-LCA (*n* = 7; ages 18–47) and *CEP290*-LCA (*n* = 3; ages 15–17) participated in the study. All subjects had complete clinical ocular examinations (Supplemental Table 1).

### Mobility device: discrimination of door on wall

**Fig. 1:**
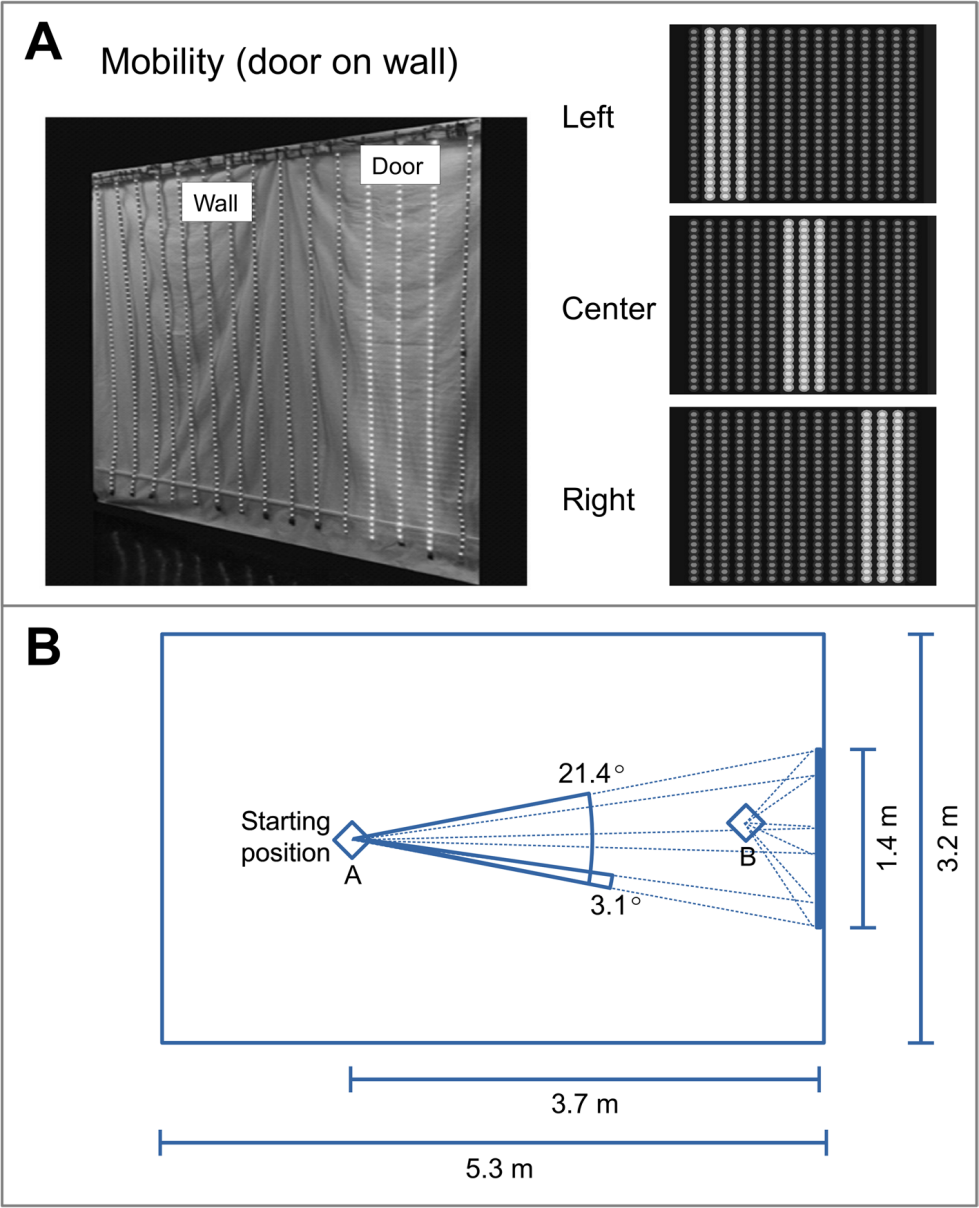
Mobility task to assay rod vision. **A** LED device and mounting used to perform the task. An illuminated pattern (‘Door’) was presented at one of three positions (right) and the subject asked to walk and touch it. **B** Room dimensions, starting position and corresponding subtended angles for the ‘wall’ and ‘door’. Dotted lines indicate the direction and angular extent of the door when viewed from the starting position (A) and from another position closer to the door (B)

We previously developed an LED-based visual orientation outcome and used it to demonstrate the severe cone dysfunction in LCA [14]. Now, we adapted the task to quantify mobility thresholds under dark-adapted conditions. A device consisting of vertical illuminated strips on a plane resembling a beaded curtain (Fig. 1 A, left) was programmed to produce a rectangular pattern target defining a ‘door’ of varying luminance that could appear at one of three positions (Fig. 1A, right). Mobility performance was evaluated by letting the subject walk from a fixed starting position with instructions to touch the door. Success was defined as the subject touching within the ‘door’ area. Ten runs were performed, and success/failure was recorded for each run. Then this process was repeated for varying levels of luminance. Tests were performed monocularly on dark-adapted eyes with dilated pupils; the fellow eye was covered with an occlusion eye patch. The door mean luminance required for successful monocular navigation at threshold for normal subjects was determined and chosen as the reference level of 0 log_10_ units (l.u.), (normal population 95% c.i. -0.72 to + 0.72). The LCA patients required more light to navigate correctly with elevated mobility thresholds compared to normal.

In more detail, the device has 15 LED strips hung with 10 cm lateral separation forming a vertical plane resembling a wall (Fig. 1A, left) that is 1.4 m wide by 2 m high, subtending 21.4° horizontally and 30.3° vertically when observed from the starting position, at 3.7 m of distance from the device and laterally centered on it (Fig. 1B). Testing was conducted in a room (5.3 × 3.2 m) with matt gray-painted walls, and a dark gray floor. The device was programmed to produce a rectangular pattern target, 3-strips (20 cm, 3.1°) wide, defining a ‘door’, surrounded by the rest of the strips defining the ‘wall’ which were not illuminated. The gaze and head direction required to find the door, and the visual field subtended by it vary as the person gets closer to the target (Fig. 1B). This configuration was chosen to mimic the way a person would approach and walk through a beaded curtain. We used green (518 nm CW, 34 nm FWHM) LEDs for the ‘door’. Further design details about the implementation of the device are provided in the Supplemental information and Supplementary Fig. 1.

### Mobility performance

Mobility performance at different scene illuminations was evaluated using two metrics: percent success of navigation over a fixed number of trials, and travel duration. The first was quantified using a three alternative forced-choice (3-AFC) design implemented by randomly presenting the ‘door’ at one of three locations (left, center or right) on the ‘wall’ (Fig. 1A, right) and letting the subject walk from the fixed starting position with instructions to touch the door. Success was defined as the subject touching no further than 5 cm from either side of the strips forming the ‘door’. The percent success and travel duration were estimated by conducting a set of ten trials. The 3-AFC paradigm requires n ≥ 7 successful travels out of 10 to reject the null hypothesis of no discrimination at the α = 0.05 level, yielding a power of 88%. This procedure was performed for several levels of luminance, starting with fully dark-adapted eyes and proceeding in increasing steps of luminance. The effective luminance was varied by PWM control of the LEDs combined with attenuating wide-field goggles (field of view approximate limits 60° superior, temporal and nasal, 45° inferior). The attenuation was varied by using different numbers of 0.6 l.u. neutral density filter sheets with approximately flat response over the spectral content of the light. The filter sheet transmittance was measured with a photometer (IL1700, International Light, Peabody, MA.) using the device LEDs as the light source. The success/not success data at each set (10 trials) of all luminance steps tested were fit individually for each eye by logistic regression with asymptotes 0.3 (guessing probability) and 1.0 (lapsing probability = 0). The threshold for successful travel was defined as the luminance corresponding to 65% success on the fitted curve. The 95% range of thresholds for the normal population was estimated as ± 1.96 * SD where SD is the total standard deviation obtained from an intercept-only mixed-effects model with Threshold as the dependent variable and Subject as random effect, using threshold values from the fitting of all control eyes, *n* = 8. The 95% confidence levels for the patient thresholds were obtained individually for each eye by parametric bootstrapping. Comparison of travel duration between normals and patients for suprathreshold scene luminances was done with a similar model (with Travel time as dependent variable) using robust estimation (robustlmm library (v. 2.4.5) [17]. All analyses were performed using R Statistical Software (v4.04) [18].

### Full-field stimulus test (FST)

FST was performed monocularly in the dark-adapted state with a dilated pupil; FST thresholds were measured on the same visit and in the same eye as the mobility thresholds. The LED-based ganzfeld system used blue and red full-field stimuli; the difference in light sensitivity between the two colors provides information regarding the photoreceptor type contributing to perception. Based on the patient’s response, a different luminance would be presented according to a predefined algorithm [6, 16].

## Results

**Fig. 2:**
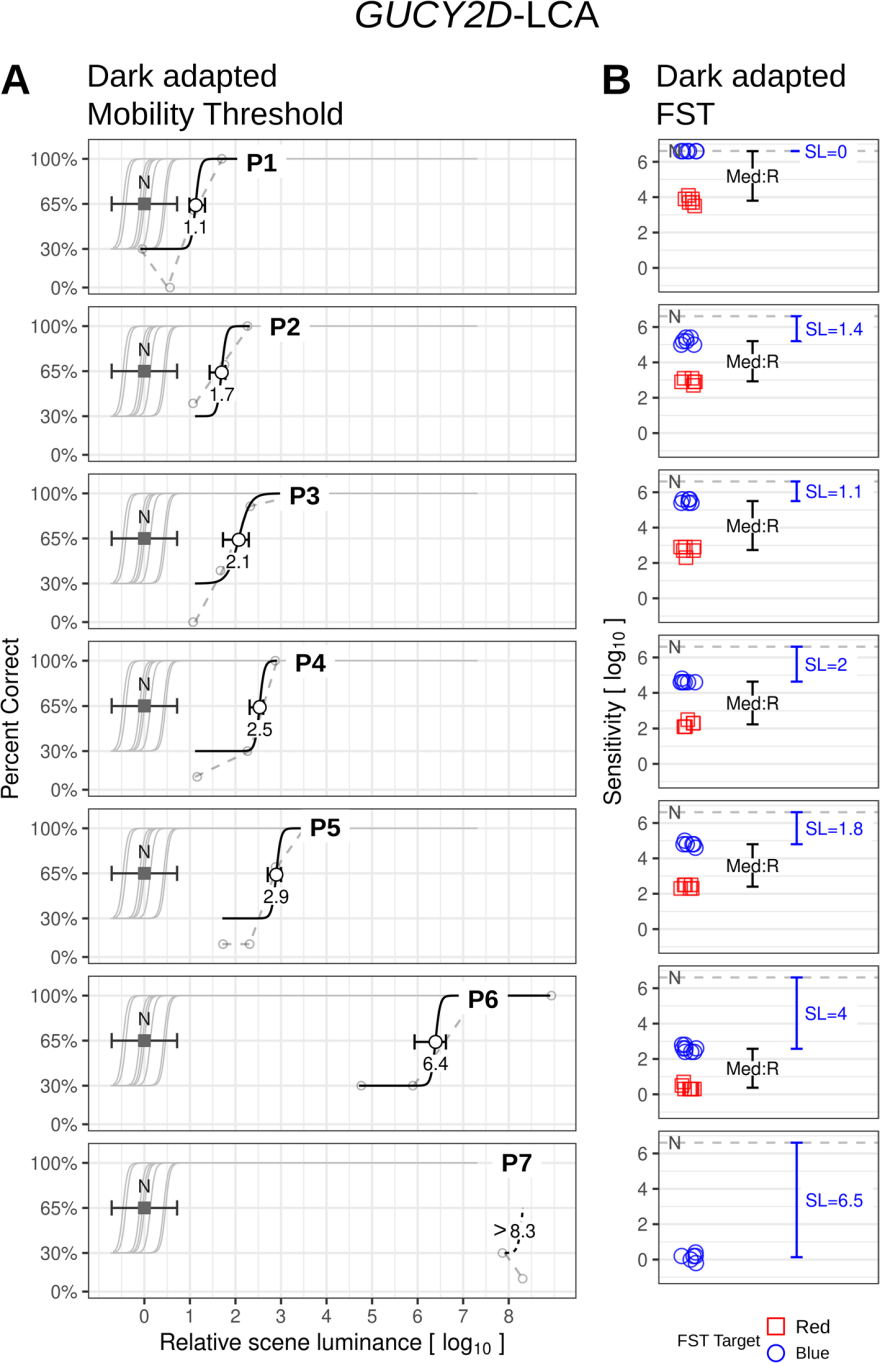
Thresholds for dark-adapted mobility in *GUCY2D* –LCA compared with data from FST results. **A** Sigmoid curves are fit to success/failure data at different target luminances, which were varied over a span of 8 l.u. in steps of 0.6 l.u. The 0 luminance level was set at the mean normal threshold (light intensity to achieve 65% success in normal subjects) and was estimated to correspond correspond to a luminance of -3.8 log scot-cd.m^-2^ originating from each LED. Patients required higher intensities to reach this percent success and thus their curves are shifted to the right. Brackets: 95% confidence interval (c.i.) for the individual patient’s threshold (circles), and for normal population range (N). Filled square, mean normal. Dashed lines joining small gray circles represent percent correct at each luminance over 10 trials. **B** Dark-adapted sensitivity as measured by FST, for blue (circles) and red (squares) targets. Mean normal threshold is indicated by a dashed line (N). The difference of sensitivities between blue and red (black brackets) indicate rod-mediation (Med:R) of both colors in the six patients with measurable values. Sensitivity loss (SL, blue brackets) in these patients range from Normal (P1) to 4 l.u. (P6). For P7, only a lower bound could be estimated for the mobility threshold due to floor effects (patient was unable to navigate at the brightest target luminance). A floor effect also precluded FST measurements for red, thus photoreceptor mediation was indeterminate. Sensitivity as measured by the blue target was very severely compromised

The dimmest scene luminance (8 l.u. attenuation from maximum) used in the current study was near the absolute functional vision threshold for normal scotopic vision. The average percent correct (identification success rate) was within the range of hypothetical probability just by chance (mean 33%; 95% confidence interval 4-63%; *n* = 10 trials; binomial), as expected from random guessing for a 3-AFC design (Fig. 2A).

LCA patients with *GUCY2D* mutations, unlike in many other molecular forms of LCA, can show considerable rod-based vision and there is a wide range from near normal to severely reduced [19, 20]. The patients in the present study illustrate such a range of results (Fig. 2). The mobility task threshold for P1 (1.1 l.u.) represents a result that is close to normal (Fig. 2A). Visual acuity of P1 was 1.04 logMAR; FST indicated rod-based mediation and normal rod sensitivity (Sensitivity Loss, SL = 0, Fig. 2B). P2 and P3 showed elevated mobility task thresholds (1.7 and 2.1 l.u., respectively), Visual acuities of P2 and P3 were 2.3 and 1.06. FST showed abnormalities (SL = 1.4 and 1.1, respectively) and FST blue minus red difference indicated rod mediation in both patients. P4 and P5 had more elevated mobility thresholds (2.5 and 2.9 l.u., respectively). Visual acuities of P4 and P5 were 1.18 and 0.9 logMAR. FST showed abnormalities (SL = 2 and 1.8 l.u., respectively) and these were also rod-mediated in both patients.

Patient P6, with a visual acuity of 1.26 logMAR, had a substantial elevation of mobility task threshold (6.4 l.u) and considerable FST loss (SL = 4 l.u.) despite retaining rod mediation. Patient P7, with light perception vision, was unable to perform the mobility task at the highest luminance level (threshold > 8.3 l.u.) and therefore exemplifies a mobility floor effect. FST sensitivity loss was greater than 6.5 l.u and photoreceptor mediation was indeterminate.

**Fig. 3:**
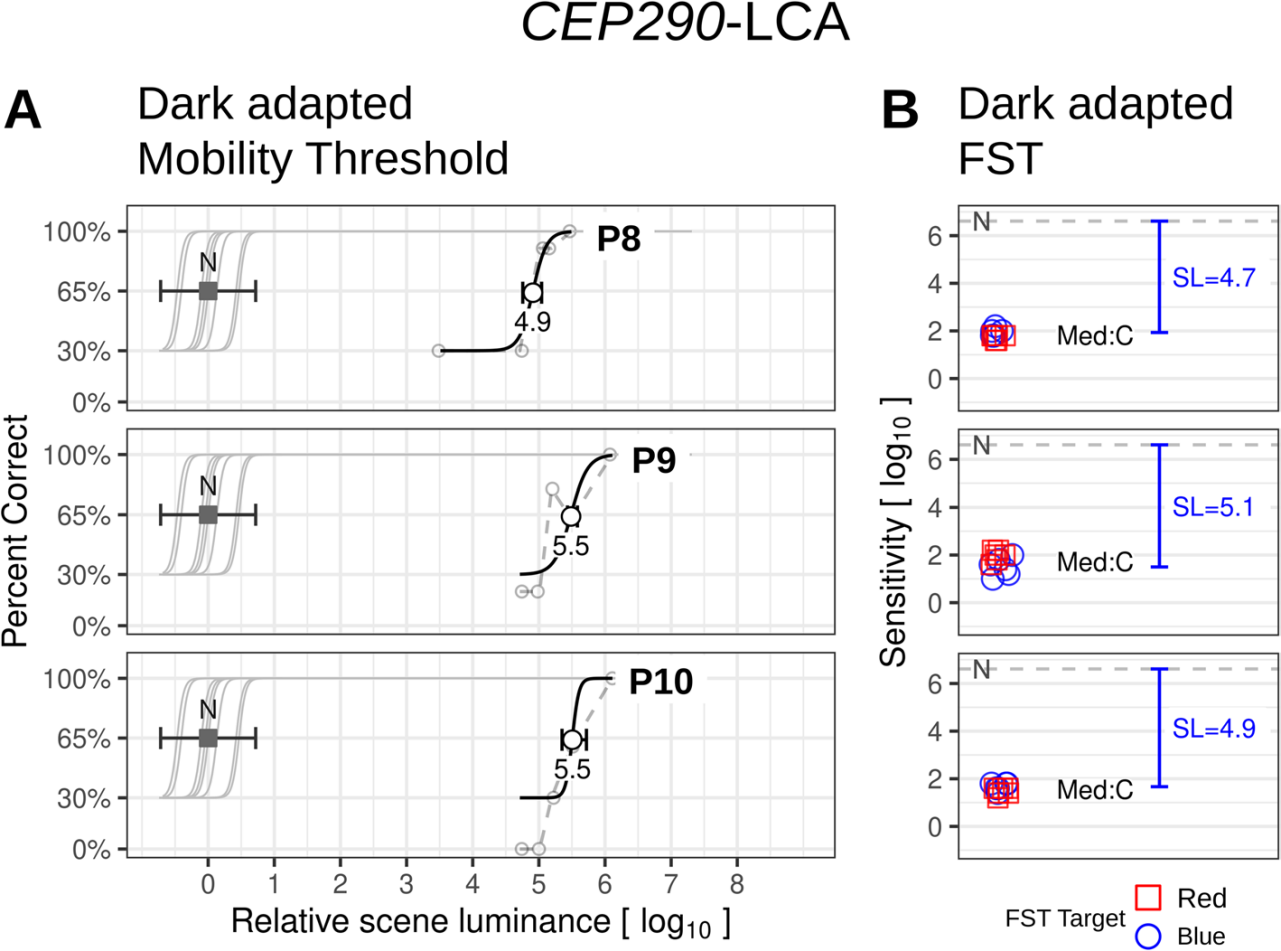
Data for *CEP290*-LCA patients shown with the same layout as Fig. 2. **A** Dark-adapted mobility thresholds, and **B** Dark-adapted sensitivity as measured by FST. In contrast with the *GUCY2D*-LCA patients, FST blue and red sensitivities were close to 0, indicating cone-mediation (Med:C). The mobility thresholds are also shifted to the right from normal and the shifts (4.9–5.5 l.u.) are consistent with the sensitivity losses by FST (blue brackets)

*CEP290*-LCA, like LCA due to *GUCY2D* mutations, is also a photoreceptor disease but there is a very different mechanism and disease expression [21–23]. Most *CEP290*-LCA patients, after an early-onset degeneration of rod photoreceptors, retain only a central island of cone photoreceptors. Chromatic FST in the three patients (P8-P10) indicates cone mediation of sensitivity and between 4.7 and 5.1 l.u. of sensitivity loss (Fig. 3B), despite a very large range of visual acuities between 0.6 and 2.0 logMAR. The mobility task thresholds do not show the wide range of results of *GUCY2D*-LCA; they range over 4.9–5.5 l.u. (Fig. 3A), which may be expected from cone vision versus normal rod vision [24, 25].

**Fig. 4:**
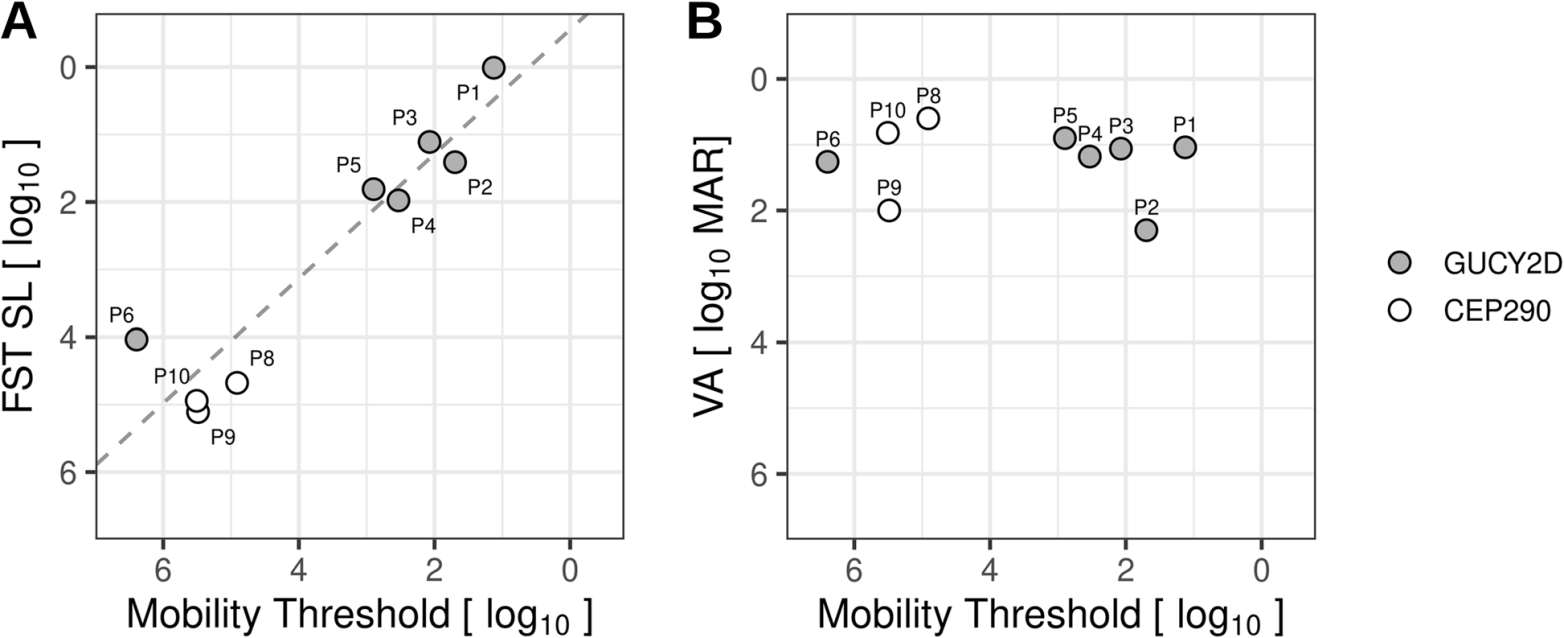
Relationship between mobility thresholds and FST sensitivity loss (**A**), and mobility thresholds and visual acuity (**B**) shown in log-log coordinates. P7 with indeterminate mobility threshold and LP visual acuity is not included in either panel. Linear regression in panel **A** (dashed line) has slope of 0.92, *r*^2^ = 0.87, *p* < 0.001, F-test. VA data (panel **B**) did not show a significant relation to mobility threshold (*p* = 0.75)

Mobility task thresholds across our cohort of LCA patients with substantial differences in disease severity showed a linear relationship with FST thresholds (Fig. 4A), but mobility was not correlated with visual acuity (Fig. 4B). In terms of transit time, in all subjects the mobility test progressed fast for suprathreshold scene luminances (0.6 l.u. brighter than the subject’s threshold), with each trial lasting a mean transit time of 5.6 s; there was no statistically significant difference between normals and patients in terms of suprathreshold transit time (*p* = 0.63). At threshold and dimmer scene luminances, transit times increased for both patients and normal subjects.

## Discussion

The clinical diagnosis of LCA includes retinopathies with a diverse set of disease mechanisms but the shared manifestation of severe visual disability [26–28]. The very low vision creates problems as to how to quantify visual function or changes thereof. Progress in the development of therapies has led to early clinical trials of LCA, and this has prompted the need to develop outcomes that are more suited to these disorders. Traditional methods to quantify changes in visual function, such as visual acuity and visual fields [1], are complicated in LCA by involuntary eye movements (nystagmus) and the resulting inability of patients to see and use a fixation target that would allow known regions of their retinas to be tested for amount of vision. One measure of visual function we developed specifically for LCA is FST, which has been used in most early phase clinical trials of LCA [11, 29–33]. FST is a psychophysical method of measuring visual function that is performed with the patient seated with their head positioned in a ganzfeld stimulator. There is a wide dynamic range of stimulus intensities that allow for severe visual loss to be probed. Different colors are used in the test to inform about which photoreceptor type is contributing to the perception. The test was designed not to depend on fixation ability [6, 16].

In addition to measures of visual function performed by a seated patient, there has been increasing recognition that ‘real-world’ functioning should also be quantified [34]. In a recent pivotal subretinal gene therapy clinical trial of *RPE65*-LCA, a mobility outcome was used [10] when visual acuity was not convincingly shown to be improved in multiple contemporaneous trials [29–33]. Results with the mobility outcome were supported by FST, deemed a standard for such trials [6, 35]. In the present study, we chose to examine patients with *GUCY2D*-LCA and *CEP290*-LCA because these forms of LCA are targets for gene or gene-based therapies [23]. *GUCY2D*-LCA patients essentially manifest a cone > rod dystrophy [36] with a notable loss of foveal cone cells and a range of rod vision results [19, 20]. There is likely to be a range of rod vision improvements in upcoming trials of *GUCY2D*-LCA which should not be disregarded. These improvements can be quantifiable by the method presented in this study. We also compared the FST results showing cone-mediated vision in *CEP290*-LCA patients to the results of the dark-adapted mobility test and there was consistency of thresholds.

The current mobility course was developed because some of the previously described commercial and investigational mobility tasks have several disadvantages. First, the dimmest ambient light conditions used in physical mobility courses (typically at 1 lx [10–12] or even 0.2 lx [19] are approximately 3 l.u. (1000 fold) brighter than normal dark-adapted threshold and thus would not be expected to detect any changes in many patients such as a subset of *GUCY2D*-LCA that retain some night vision [19, 20]. The mobility threshold test described herein covers the full dynamic range of human night vision. Second, several other mobility tasks conflate spatial vision, visual field and light sensitivity [10, 11]. The mobility threshold test we describe in the present study had a target subtending a large visual angle, expected to be visible over a very large range of visual acuity; appropriately, the current results showed no correlation of mobility thresholds with visual acuity.

Given a future pivotal trial of gene therapy in *GUCY2D*-LCA, we recommend a cone-based outcome such as standard visual acuity or a cone-specific mobility task [14] but also a ‘real-world’ outcome evaluating mobility at absolute dark-adapted threshold such as the one described herein. This should suffice to understand functional vision consequences if only rod photoreceptor phototransduction was affected in a positive way by a therapy.

## Conclusions

Recent progress in the understanding of the molecular basis and disease mechanisms of severe childhood blindness has led to natural history studies and clinical trials of treatment of these disorders. Night vision in these patients can be impaired but it is not readily measured, especially with a ‘real-world’ task. We addressed this need and developed a mobility task to determine thresholds for vision in the dark-adapted state. Two cohorts of patients with childhood blindness and different genotypes were tested with the novel method and results compared with results in the same patients using FST, a desktop tool considered a standard night vision assay. Results with the two different assays compared favorably, with dark-adapted mobility task thresholds showing a monotonic relationship with FST thresholds.

## Supplementary Information

**Additional file 1: Supplemental Figure 1.** User interface for the android app used to conduct the test. Controls are rendered in red over a black background to minimize light interference in the room. Downwards from the top, they handle bluetooth channel connection / disconnection to the wall device, manual or random selection of left / center / right position of the door, its intensity both continually (slider) and using presets, volume setting for the optional sound to be issued at start of presentation, and a start/stop/reset timer.

**Additional file 2: Supplemental Table 1.** Clinical Characteristics of LCA Patients.

**Additional file 3.**

## Data Availability

The datasets during and/or analysed during the current study are available from the corresponding author on reasonable request.

## Abbreviations

CEP290: centrosomal protein, 290 kD
CW: Center wavelength
FWHM: Full-width at half-maximum
GUCY2D: guanylate cyclase 2D, membrane (retina-specific)
LCA: Leber congenital amaurosis
LED: Light emitting diode
PWM: Pulse-width modulation
RPE65: retinal pigment epithelium-specific 65 kDa
