# Supplementary figures and images for: Mobility test to assess functional vision in dark-adapted patients with Leber congenital amaurosis
Source: BMC Ophthalmol. 2022 Jun 14;22:266. doi: 10.1186/s12886-022-02475-y (PMC9195222; doi:10.1186/s12886-022-02475-y)

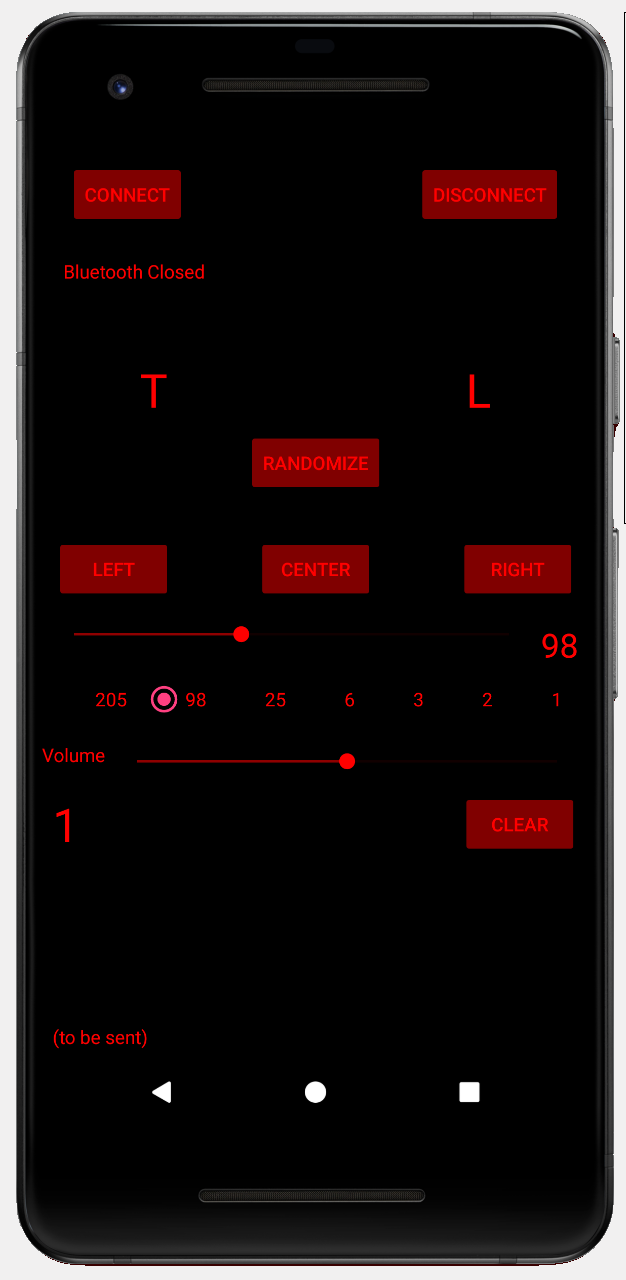

Supplement: Supplementary file 1 — Additional file 1: Supplemental Figure 1. User interface for the android app used to conduct the test. Controls are rendered in red over a black background to minimize light interference in the room. Downwards from the top, they handle bluetooth channel connection / disconnection to the wall device, manual or random selection of left / center / right position of the door, its intensity both continually (slider) and using presets, volume setting for the optional sound to be issued at start of presentation, and a start/stop/reset timer. [file 12886_2022_2475_MOESM1_ESM.tiff]
