# Supplementary material for: Mobility test to assess functional vision in dark-adapted patients with Leber congenital amaurosis
Source: BMC Ophthalmol. 2022 Jun 14;22:266. doi: 10.1186/s12886-022-02475-y (PMC9195222; doi:10.1186/s12886-022-02475-y)
Supplement: Supplementary file 2 — Additional file 2: Supplemental Table 1. Clinical Characteristics of LCA Patients. [file 12886_2022_2475_MOESM2_ESM.docx]

**Supplemental Table 1.** Clinical Characteristics of LCA Patients

| **Gene** | **Age Group (years)** | **Patient ID/Sex** | **BCVA (logMAR)** |
| --- | --- | --- | --- |
|  |  |  |  |
| *GUCY2D* | 10-19 | P1/F | 1.04 |
|  |  | P3/M | 1.06 |
|  | 20-29 | P5/F | 0.90 |
|  | 30-39 | P6/F | 1.26 |
|  |  | P7/M | 3.90 |
|  |  | P4/M | 1.18 |
|  | 40-49 | P2/F | 2.30 |
| *CEP290* | 10-19 | P8/F | 0.60 |
|  |  | P9/F | 2.00 |
|  |  | P10/F | 0.82 |
