# Supplementary material for: Mobility test to assess functional vision in dark-adapted patients with Leber congenital amaurosis
Source: BMC Ophthalmol. 2022 Jun 14;22:266. doi: 10.1186/s12886-022-02475-y (PMC9195222; doi:10.1186/s12886-022-02475-y)
Supplement: Supplementary file 3 — Additional file 3. [file 12886_2022_2475_MOESM3_ESM.docx]

**Supplemental information**

**Implementation details of the dark-adapted mobility method**

The device used was described in [14], but we are providing additional design details to aid in replication. The odd- and even- numbered LED strips (Neopixel RGB-PID1460, Adafruit Industries, NY) are grouped and each group fed by a separate medical-grade regulated power supply. Power for each strip 5-V supply rail is injected from the top. The connection to the corresponding power supply uses individual wiring of approximately the same length. This power distribution minimizes power-line cross-talk between strips so that the light intensity output from one strip is minimally affected by the on/off state of the others. The shorter length of the strips assures that the voltage drop affecting pixels located at the bottom is small and compensable by the constant-current drivers integrated in the pixel devices. The strip architecture permits updating the whole set of LEDs in the wall serially from a

single controller channel, with updates flowing sequentially through each of the 600 LED units. This would require daisy-chaining the strip control lines and introduce a non-uniform delay for pixel updates, so we opted for an alternative data distribution using dedicated controller channels driving each strip data line in parallel. This distribution scheme permits updates to arrive with lower differential delay to the head of each 60-pixel strip so the updating of the whole wall appears simultaneous. The LED devices are not flexible in terms of control signal timing so we used a microprocessor controller (Arduino Mega, Ivrea, Italy) able to handle the serial channels on multiple I/O pins. The microprocessor firmware initializes all pixels at startup, and waits for short commands from a serial port. A full wall update is triggered at full reception of each command. The microprocessor serial port is connected to a bluetooth controller (Bluefruit EZ-link, Adafruit Industries, NY) that relays commands transparently. This controller is paired with a handheld device (an android smartphone or tablet) running the user interface app programmed using the AndroidStudio framework and standard android platform libraries (Supplemental Figure 1). The app can produce a warning sound at start of the trial, using the android device speaker. As this device is physically located at the back of the room, no auditory clue is given to the subject with regards to wall or door location. A manual timer is also added to measure transit timing over the mobility trial. The timer is triggered, stopped and reset by sequentially pressing one of the hardware switches on the side of the device.
